# Prognostic value of expression of molecular markers in adenoid cystic cancer of the salivary glands compared with lymph node metastasis: a retrospective study

**DOI:** 10.1186/1477-7819-10-266

**Published:** 2012-12-11

**Authors:** Seok Ki Lee, Min Su Kwon, Yoon Se Lee, Seung-Ho Choi, Sang Yoon Kim, Kyoung Ja Cho, Soon Yuhl Nam

**Affiliations:** 1 Department of Otolaryngology, Kangwon National University, College of Medicine, 192-1 Hyoza 2-Dong, Chuncheon, Kangwon, 200-701, South Korea; 2 Department of Otolaryngology, Asan Medical Center, University of Ulsan, College of Medicine, 388-1 Pungnap-2dong, Songpa-gu, Seoul, 138-736, South Korea; 3 Department of Otorhinolaryngology-Head and Neck Surgery, Research Institute for Convergence of Biomedical Science and Technology, Pusan National University Yangsan Hospital, Beomeo-ri, Mulgeum-eup, Yangsan, Gyeongsangnam-do, 626-770, Republic of Korea; 4 Department of Pathology, Asan Medical Center, University of Ulsan, College of Medicine, 388-1 Pungnap-2dong, Songpa-gu, Seoul, 138-736, South Korea

**Keywords:** Salivary gland cancer, Adenoid cystic cancer, c-kit, Epithelial growth factor receptor, Vascular endothelial growth factor, Lymph node metastasis

## Abstract

**Background:**

Adenoid cystic cancer arising in the salivary glands has distinctive features such as perineural invasion, distant metastasis, and a variable prognosis. In salivary gland cancer, c-kit, EGFR, and VEGF are representative molecular markers that may predict remnant and recurrent tumors. In this study, the expression of c-kit, EGFR, and VEGF in adenoid cystic cancer was evaluated, and the relationships between the expression of these markers and the clinical findings were investigated.

**Methods:**

The medical records of 48 patients who were treated for parotid adenoid cystic cancer from January 1990 to January 2006 were reviewed. The tumor location, size, histological subtypes, perineural invasion, the resected margin status, and lymph node metastasis were assessed. Immunohistochemical staining and semiquantitative analysis of c-kit, EGFR and VEGF were performed. The relationship between the expression of each marker and the clinicopathological factors were analyzed.

**Results:**

Positive c-kit immunostaining was present in 45 patients (94%), with weak positivity (+1) in 23, moderate positivity (+2) in 19 and strong positivity (+3) in three. Positive EGFR immunostaining was observed in 27 (56%), with weak positivity (+1) in 19 and moderate positivity (+2) in eight with no strong positive staining. Positive VEGF immunostaining was present in 42 patients (88%) with weak positivity (+1) in 12, moderate positivity (+2) in 17, and strong positivity (+3) in 13. Only the expression of VEGF was significantly higher in parotid gland tumors than in any other gland (*P* = 0.032). Marginal involvement was associated with strong VEGF expression (*P* = 0.02). No marker was significantly correlated with recurrence or the survival rate. Lymph node status was related to the survival rate.

**Conclusions:**

The expression of c-kit, EGRF, and VEGF had no predictive value for recurrence or the prognosis of adenoid cystic cancer. Only the lymph node status was related to the prognosis.

## Background

Adenoid cystic carcinoma is a relatively rare tumor in the parotid gland with a 10% incidence, compared with a 30% incidence in the minor salivary glands. Histologically, this tumor shows higher rates of local growth, perineural invasion and distant metastasis. Many cases of delayed recurrence after definite treatment have been reported, indicating the difficulty of treating and controlling this tumor. Factors related to the recurrence and prognosis of adenoid cystic carcinoma include tumor stage, histological classification, status of the resected tumor margin, lymph node metastasis, and perineural invasion [1-3]. Investigations of the molecular markers of this tumor and the development of targeted therapy are currently ongoing. Ki-67, p53, bcl-2, epidermal growth factor receptor (EGFR), human epidermal growth receptor-2 (HER2), proliferating cell nuclear antigen (PCNA), and Runt-related transcription factor-3 (RNUX3) are representative markers of adenoid cystic carcinoma [4-6]. The inhibition of vascular endothelial growth factor (VEGF) has also been reported to reduce local recurrence and distant metastasis [7].

The c-kit protein is a transmembrane receptor with a tyrosine kinase function. The overexpression of c-kit has been reported in various malignant tumors [8,9], including adenoid cystic carcinoma and some salivary gland tumors [10,11]. However, there is little information on the prognostic value of c-kit, and the therapeutic efficacy of a tyrosine kinase inhibitor (imatinib) is not well established [12]. EGFR is a transmembrane receptor found in epithelial cancers such as breast, lung, bladder, ovary, prostate, and head and neck cancers [3,13]. The overexpression of EGFR in head and neck cancer is associated with an advanced stage, lymph node metastasis, low survival rate, and poor response to radiation therapy [14,15]. Although many studies on the role of EGFR in salivary gland tumors have been reported, there is some debate about its prognostic significance [16]. VEGF is a major vascular formative factor in the process of epithelial carcinogenesis and tumor metastasis. It selectively functions in the vascular endothelial tissue and is expressed in various organs such as lung, breast and gastrointestinal organs [17]. The overexpression of VEGF is involved in perineural invasion and recurrence, and it heralds a poor survival rate [18]. In an animal study, the inhibition of VEGF expression reduced growth and distant metastasis of adenoid cystic cancer [19]. Clinical trials with a single inhibitor (imatinib for c-kit; trastuzumab or cetuximab for ErbB1 and ErbB2) have shown low response rates in salivary gland cancer [20]. Intriguingly, concomitant inhibition of EGF and VEGF reduced the growth and metastasis of adenoid cystic carcinoma in an animal model [19]. EGFR overexpression and the absence of c-kit expression are negative prognostic factors in adenoid cystic cancer [21]. Based on these reports, we hypothesized that the elucidation of the diverse changes in these proteins would improve treatment outcomes. In the present study, we evaluated the relationship between c-kit, EGFR, and VEGF expression and the survival or local control rate of adenoid cystic carcinoma.

## Methods

### Patients and treatment

This retrospective study using medical chart reviews was performed after approved by the institutional review board of Asan Medical Center. Written informed consent was obtained from the patients for publication of this report and any accompanying images. We reviewed the medical records of 48 patients who were diagnosed with and treated for salivary adenoid cystic carcinoma from January 1990 to January 2006 at a single institute. The presence of lymph node metastasis was preoperatively evaluated by neck ultrasonography (US), computed tomography (CT), and magnetic resonance imaging (MRI). Tumor locations were divided into the submandibular, parotid, and minor salivary glands. Distant metastasis was identified with Tc^99m^ bone scanning and F^18^-flurodeoxyglucose positron emission tomography (F^18^-FDG PET). We investigated tumor size, the histological pattern, the presence of perineural invasion, invasion of surgical margins, and lymph node metastasis according to the pathology reports. The histological patterns were classified as tubular, cribriform, and solid types on the basis of the WHO classification (2005). Marginal involvement included microscopic and macroscopic invasion of the margin of the specimen. When lymph node metastasis was confirmed on preoperative fine needle aspiration cytology with or without US, therapeutic neck dissection from level I to V was performed. Selective neck dissection, including levels I to III, was performed in cases of clinical T3, T4 or suspicious findings on radiology. Lymph node metastasis was defined when confirmed histologically after surgery. A close resection margin (<<1 cm from the tumor), an unclear margin, a perineural invasion, and an advanced stage (III, IV) were indications for adjuvant radiation therapy. We also checked for local recurrence, distant metastasis, and survival after treatment [22,23]. The observation period was defined from the end point of treatment to the most recent follow-up date.

### Tissue microarray blocks

Two tissue microarray blocks were made from the formalin-fixed, paraffin-embedded tissue of the 48 patients with adenoid cystic carcinoma. Each slide was reviewed under a microscope, and representative areas were selected for immunohistochemistry. These were marked on the slides stained with hematoxylin and eosin, and their corresponding areas on each block were punched using a tissue cylinder with a 0.6 mm diameter. These cores were transferred to the recipient blocks using a tissue-arraying instrument (Beecher Instruments, Silver Spring, MD, USA). Considering the heterogeneity of the tumor, the samples were arrayed in duplicate to avoid tissue loss.

### Immunohistochemistry

Immunohistochemical staining for c-kit, EGFR, and VEGF was performed using a Benchmark automatic immunostaining device (Ventana Medical Systems, Tucson, AZ, USA). Tissue sections (4 μm thick) were deparaffinized in 10% xylene, dehydrated in 100%, 95%, and 75% ethanol solutions, and washed with distilled water. For antigen retrieval, the sections were immersed in 10 mM citrate buffer (pH 6.0) and microwaved for 10 min. Endogenous peroxidases activities and nonspecific antigens were eliminated with 3% hydrogen peroxide in methanol and AB blocker (Roche, Basel, Switzerland). The sections were incubated with primary antibodies against c-kit (CD117, 1:400 dilution; DAKO, Glostrup, Denmark), EGFR (1:500 dilution; DAKO), and VEGF (1:100 dilution; Zymed, South San Francisco, CA, USA). Immunodetection was performed with biotinylated anti-mouse immunoglobulin followed by peroxidase-labeled streptavidin, using a labeled streptavidin-biotin kit (DAKO). As a chromogen, 3,3’-diaminobenzidine was used, and the sections were counterstained with Harris hematoxylin. The intensity of the immunohistochemical staining of c-kit (membranous/cytoplasmic), EGFR (membranous), and VEGF (cytoplasmic) was semiquantitatively scored as 0 (reactivity in <5% of the tumor cells), 1+ (reactivity 5% - 1/3 of the tumor cells), 2+ (reactivity in 1/3 to 2/3 of the tumor cells), or 3+ (reactivity >2/3 of the tumor cells). Cases with scores of 2+ and 3+ were regarded as positive.

### Statistical analysis

SPSS software (version 14.0; SPSS Inc., Chicago, IL, USA) was used for the statistical analysis. To identify relationships between clinical factors and immunostaining of markers, Fisher’s exact test was used for categorical data and the Mann–Whitney *U* test was used for continuous variables. Univariate and multivariate analyses using Cox’s proportional hazard model were performed with a backward, stepwise elimination approach. At each step, the least significant factor with *P* >0.10 was eliminated, and each remaining factor in the model was reassessed. The limit for including factors was set at 20%. To simplify binary logistic regression analyses, we divided the data into two groups based on immunoreactivity. The Kaplan-Meier method was used to analyze survival rates. Values of *P* below 0.05 indicated statistical significance.

## Results

### Study population

Forty-eight patients (19 males and 29 females) with adenoid cystic cancer in the salivary gland were enrolled. The average patient age was 49.7 years (± 21.9). The numbers of tumors that involved the submandibular gland, parotid gland, or minor salivary gland were 12 (25%), 18 (38%), and 18 (38%), respectively. The average tumor size was 2.6 cm (± 2.2). Twenty-seven cases were classified into three groups (tubular, two cases (4%); cribriform, 18 cases (38%); solid, seven cases (15%)) according to the histology, and 21 cases (44%) had no records or had unclear tumor types. Perineural invasion and cervical lymph node metastasis were identified in 22 (56%) and six (13%) cases, respectively. Twelve patients underwent PET, and the average standard uptake value in the primary lesion was 3.0 (± 2.1). Ten patients (21%) underwent only surgical management, while 32 (67%) received adjuvant radiation therapy after surgery. Concurrent chemoradiation therapy following surgery was administered in six cases (14%) (Table 1).

**Table 1:** Characteristics of patients and tumors

**Characteristics**	**Category**	**Number of patients (%)**
Sex	Male	19 (40)
	Female	29 (60)
Site	Submandibular	12 (25)
	Parotid	18 (38)
	Minor	18 (38)
Histology	Tubular	2 (4)
	Cribriform	18 (38)
	Solid	7 (15)
	Indeterminate	21 (44)
Perineural invasion	Negative	26 (54)
	Positive	22 (46)
Resection margin	Negative	21 (44)
	Positive	27 (56)
Lymph node involvement	Negative	42 (88)
	Positive	6 (13)
Treatment	Surgery only	10 (21)
	Surgery + RT	32 (67)
	Surgery + CCRT	6 (13)
		Mean (± SD)
Age (years)		49.7 (± 14.2)
Tumor size (cm)		2.5 (± 1.2)
PET (SUV)		3.0 (± 1.2)

CCRT, concurrent chemoradiation therapy; PET, positron emission tomography; RT, radiation therapy; SUV, standard uptake value.

### Immunohistological staining

**Figure 1:**
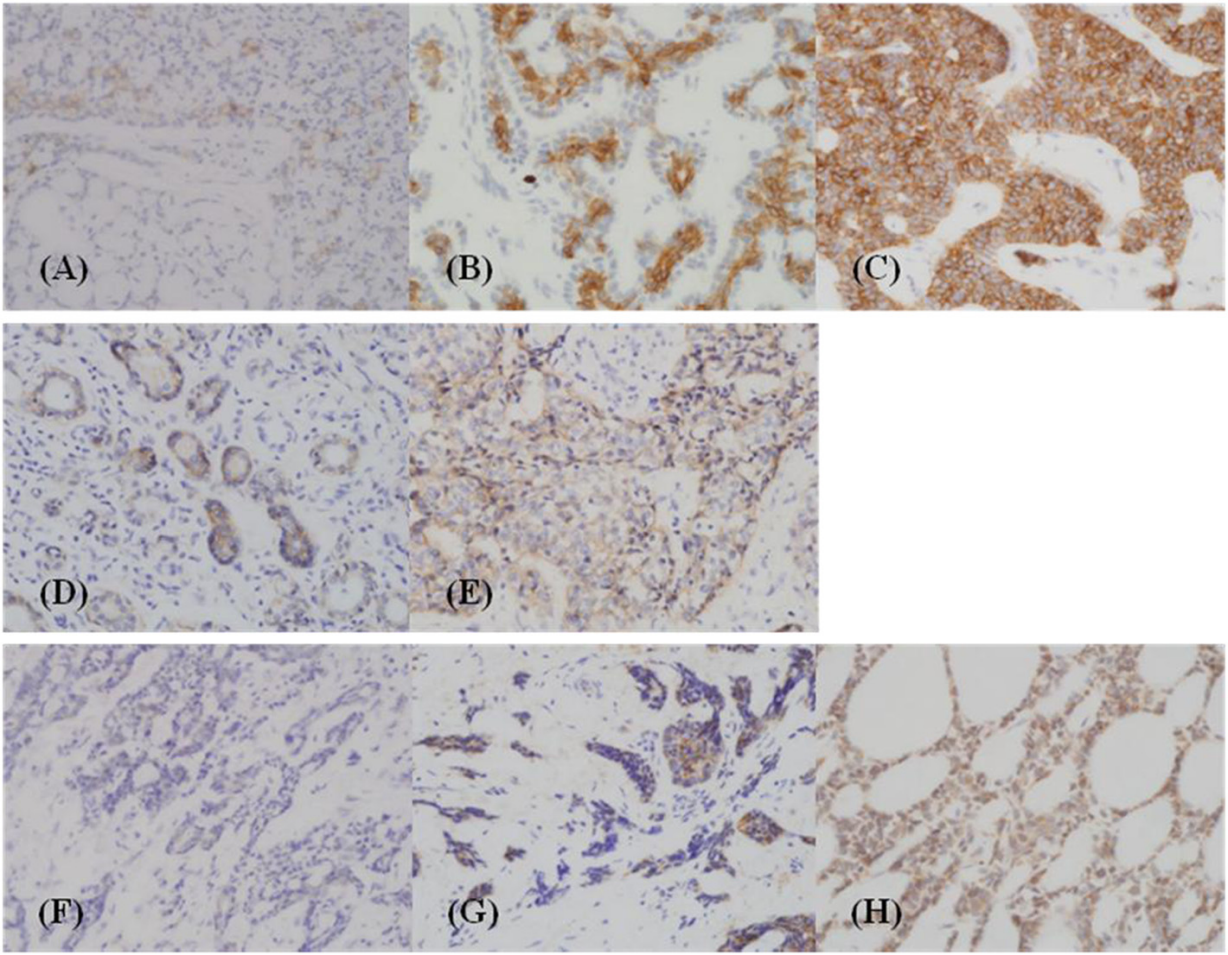
**Immunohistochemical staining of adenoid cystic carcinomas of the salivary gland.** C-kit (CD 117) expression of weakly (1+, **A**), moderately (2+, **B**), and strongly positive (3+, **C**) cases; EGFR (epidermal growth factor receptor) expression of weakly **(D)** and moderately positive **(E)** cases; VEGF (vascular endothelial growth factor) expression of weakly **(F)**, moderately **(G)**, and strongly positive **(H)** cases. Expressions of c-kit (membranous/cytoplasmic), EGFR (membranous), and VEGF (cytoplasmic) were scored as follows: 0, reactivity in <5% of tumor cells; 1+, reactivity in 5% -1/3 of tumor cells; 2+, reactivity in 1/3 to 2/3 of tumor cells; 3+, reactivity >2/3 of tumor cells.

Forty-five cases (94%) showed c-kit immunoreactivity, with 23 weakly positive, 19 moderately positive, and three strongly positive cases. For EGFR, 27 cases (56%) were positive, comprising 19 weakly positive and eight moderately positive cases; no strongly positive cases were observed. Forty-two cases (88%) showed positive immunoreactivity for VEGF: 12 were weakly positive, 17 were moderately positive, and 13 were strongly positive (Figure 1). The patients were divided into two groups, a negative or weakly positive group and a more-than-moderately positive group, according to the staining intensity for c-kit and VEGF. For EGFR, patients were divided into negative and positive groups. There was no significant correlation between the staining intensity for each marker (c-kit and EGFR, *P* = 0.422; EGFR and VEGF, *P* = 0.499; VEGF and c-kit, *P* = 0.454). VEGF showed stronger expression in the parotid gland (*P* = 0.032) than in the other sites, and marginal invasion was frequent in cases with high VEGF expression (*P* = 0.02). The other makers showed no notable trends (Table 2).

**Table 2:** Correlation between expression of tumor markers and clinical factors

		**c-kit**			**EGFR**			**VEGF**		
**Clinical factor**		**0, 1+**	**2+, 3+**	***P***	**0**	**1+, 2+**	***P***	**0, 1+**	**2+, 3+**	***P***
Sex	Male	12	7	0.312	9	10	0.683	6	13	0.493
	Female	14	5		12	17		12	17	
Site	Submandibular gland	5	7	0.149	6	6	0.53	1	11	0.032
	Parotid gland	8	10		6	12		7	11	
	Minor salivary gland	13	5		9	9		10	8	
Histology	Tubular	1	1	0.961	0	2	0.633	0	2	0.8
	Cribriform	9	9		7	11		7	11	
	Solid	4	3		4	3		2	5	
	Indeterminate	12	9		10	11		9	12	
Perineural invasion	Negative	15	11	0.594	12	14	0.715	8	18	0.295
	Positive	11	11		9	13		10	12	
Resection margin	Negative	13	8	0.343	7	14	0.199	11	10	0.02
	Positive	13	14		14	13		7	20	
Lymph node involvement	Negative	24	18	0.392	17	4	0.383	16	26	1
	Positive	2	4		25	2		2	4	

EGFR, epidermal growth factor receptor; VEGF, vascular endothelial growth factor.

### Recurrence after treatment

The mean follow-up period was 70.9 months (range, 4.0 to 159.1 months). There were 19 patients (39.6%) with recurrence, and the average time until detection of recurrence was 24.9 months (range, 2.0 to 97.6 months). Four cases showed local recurrence, 10 showed distant metastasis (lung, nine cases; multiple, one case), and five showed both local and distant metastasis. Lymph node metastasis was closely related to recurrence after the initial treatment (*P* <0.001). However, recurrence was not associated with the staining intensity of c-kit (*P* = 0.987), EGFR (*P* = 0.747), or VEGF (*P* = 0.927) (Table 3).

**Table 3:** Effect of tumor markers and clinical factors on tumor recurrence

**Marker/factor**		**HR**	**95% CI**	***P***
c-kit	0, 1+	1	0.402 - 2.45	0.987
	2+, 3+	0.993		
EGFR	0	0.862	0.349 - 2.129	0.747
	1+, 2+	1		
VEGF	0, 1+	0.958	0.386 - 2.377	0.927
	2+, 3+	1		
Sex	Male	1	0.195 - 1.212	0.122
	Female	0.486		
Site	Submandibular gland	1		
	Parotid gland	0.392	0.18 - 1.45	0.153
	Minor salivary gland	0.81	0.266 - 2.468	0.71
Histology	Tubular	1		
	Cribriform	0.513	0.061 - 4.283	0.538
	Solid	3.727	0.423 - 32.813	0.236
	Indeterminate	0.446	0.053 - 3.781	0.459
Perineural invasion	Negative	1	0.384 - 2.405	0.932
	Positive	0.961		
Resection margin	Negative	1	0.224 - 1.406	0.217
	Positive	0.561		
Lymph node involvement	Negative	1	2.178 - 19.982	<0.001
	Positive	6.597		
Treatment	Surgery only	1		
	Surgery + RT	0.89	0.192 - 4.122	0.881
	Surgery + CCRT	4.002	0.793 - 20.197	0.093
Age (years)		0.949	0.902 - 0.998	0.071
Tumor size (cm)		0.864	0.566 - 1.318	0.498

CCRT, concurrent chemoradiation therapy; CI, confidence interval; EGFR, epidermal growth factor receptor; HR, hazard ratio; RT, radiation therapy; VEGF, vascular endothelial growth factor.

### Survival rate

**Figure 2:**
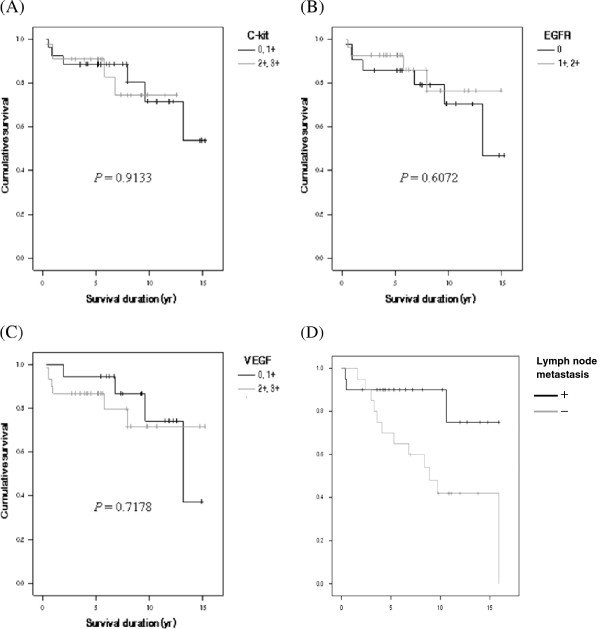
**Overall survival curves of the patients according to (A) c-kit, (B) epidermal growth factor receptor (EGFR), (C) vascular endothelial growth factor (VEGF) expression, and (D) lymph node metastasis.** Survival curves were calculated using the Kaplan-Meier method and analyzed by the log-rank test.

Eleven patients died of the disease, and the mean survival period was 38.0 months (range, 4.4 to 144.2 months). The five-year survival rate was 88.2%, and the complete remission rate was 72.9%. No marker staining intensity was associated with a difference in survival rate based on the Kaplan-Meier survival curve, whereas lymph node metastasis was significantly related to survival rate (Figure 2). Using Cox’s proportional hazard model, we analyzed the factors that might have influenced the survival rate, and only lymph node metastasis showed a close relationship to the survival rate (*P* = 0.049). The expression of c-kit (*P* = 0.864), EGFR (*P* = 0.716), and VEGF (*P* = 0.198) did not show a close relationship with the survival rate (Table 4).

**Table 4:** Effect of tumor markers and clinical factors on survival

**Marker/factor**		**HR**	**95% CI**	***P***
c-kit	0, 1+	1	0.297 - 4.251	0.864
	2+, 3+	1.124		
EGFR	0	1	0.206 - 2.957	0.716
	1+, 2+	0.781		
VEGF	0, 1+	1	0.624 - 11.392	0.198
	2+, 3+	2.623		
Sex	Male	1	0.01 - 0.639	0.17
	Female	0.8		
Site	Submandibular gland	1		
	Parotid gland	0		0.995
	Minor salivary gland	0.33	0.07 - 1.563	0.162
Perineural invasion	Negative	1	0.491- ~ 7.02	0.362
	Positive	1.856		
Resection margin	Negative	1	0.173 - 2.505	0.54
	Positive	0.659		
Lymph node involvement	Negative	1	0.791 - 14.06	0.049
	Positive	4.362		
Treatment	Surgery only	1		
	Surgery + RT	0.224	0.033 - 1.508	0.124
	Surgery + CCRT	0.831	0.113 - 6.127	0.856
Age (year)	<45	1		
	≥45	1.078	1.016 ~ 1.143	0.103
Tumor size (cm)	≤2	1		
	>2	0.838	0.452 ~ 1.554	0.575

CCRT, concurrent chemoradiation therapy; CI, confidence interval; EGFR, epidermal growth factor receptor; HR, hazard ratio; RT, radiation therapy; VEGF, vascular endothelial growth factor.

## Discussion

The low response rate of adenoid cystic cancer to tyrosine kinase inhibitors and EGFR inhibitors indicates that no single molecule is dominant in tumor invasion and spread of this cancer [19-21,24]. The class III receptor tyrosine kinase and c-kit is important for normal hematopoiesis, melanin production and reproductive cell production. Imatinib mesylate (Gleevec™), a tyrosine kinase inhibitor, is very effective in treating chronic myeloid leukemia and show good efficacy for the treatment of advanced gastrointestinal tumors with positive c-kit expression [24]. The expression of c-kit in salivary adenoid cystic cancer has been variably reported as 78% to 100%. The high c-kit expression in adenoid cystic cancer suggests that this cancer may respond to treatment with imatinib mesylate, although studies with imatinib have not shown consistent results [12,25]. The relationship between c-kit expression and prognosis has been controversial, although the expression of c-kit was stronger in the tubular or cribriform type than in the solid type [21,26]. The discrepancy might have been caused by differences in staining techniques and the lack of a standard method for evaluating staining intensity. In the present investigation, 94% of the tumors expressed c-kit, but no significant relationship with other clinical variables or prognosis was identified.

EGF is found in the primary duct and terminal buds, and its distribution and density decrease with aging [27]. EGFR is present in salivary gland tumors, with expression reported in 0% to 85% of adenoid cystic cancers [27]. In the present study, 56% of the tumors were positive for EGFR. The overexpression of EGFR is reportedly related to a poor prognosis in salivary gland cancer [21], which is different from our results. VEGF promotes vascular proliferation in tumors from peripheral tissue and enhances vessel permeability with a selective effect on vascular endothelial cells. VEGF expression is found in various tumors, such as those of esophageal cancer, small cell lung cancer, thyroid cancer, breast cancer, and cervical cancer [28], as well as head and neck cancer. VEGF expression is associated with cervical lymph node metastasis, higher cancer stage, perineural invasion, recurrence, and low survival [18,28]. Higher VEGF expression was found in solid tumors compared with tubular or cribriform types, and the survival rate decreased with higher VEGF expression [29]. In the present study, there was no relationship between high VEGF expression and lymph node metastasis, recurrence, or survival, contrary to our expectations. This was possibly, attributable to the subjective assessment of the differences in immunostaining and the semiquantitative analysis. A few investigations on treatments targeting VEGF in salivary gland cancer have shown partial remission, suggesting the targeting of VEGF for the management of adenoid cystic cancer [30]. Negative results regarding the prognostic usefulness of molecular markers in the present study might have been due to the threshold criteria used for positive EGFR, c-kit, and VEGF on immunoreactivity [21]. The availability of normal tissue as a negative control would have helped in scoring positive immunochemical staining.

Histological differentiation in adenoid cystic cancer is variable and reflects its malignant potential [31]. Indeterminate cases comprised 44% of all cases in the present study, and most of these were mixed type with no predominant type. This result was attributable to a lack of detailed pathologic reports on subtypes, which is a limitation of the present retrospective study. In addition, tumor size and involved sites were not recorded definitively enough to be analyzed. Lymph node metastasis alone was significantly associated with the prognosis. Previous studies have reported that perineural invasion had strong relationships to distant metastasis and the survival rate [32,33]. These relationships were not significant in the present investigation because of the small number of cases. In earlier studies, cervical lymph node metastasis was present in 20% of adenoid cystic cancers and was related to a high recurrence rate and low survival rate [34,35]. A similar pattern of metastasis was observed in the present study. Distant metastasis was observed in 10 cases, with the lung being the most frequently involved single site (*n* = 9). Multiple sites (lung, bone, and brain) were involved in one case.

Recurrence of adenoid cystic cancer has been found usually within 56 months. Death occurred within three years after the detection of distant metastasis in 54%, whereas survival in excess of 10 years was recorded in 10% of the patients [36,37]. Considering that late recurrence is characteristic of adenoid cystic cancer, the analysis of recurrence during a short follow-up period is another limitation of the present study.

Lymph node metastasis is a key step in the development of metastasis and is a determining factor of prognosis. Traditionally, it has been thought that tumors spread to lymph nodes by direct extension in an orderly, defined manner based on mechanical considerations and transverse lymphatics [38]. However, lymph node metastasis cannot explain local recurrence, distant metastasis, or poor treatment outcomes. Based on clinical and laboratory research, Fisher has proposed that biological, rather than anatomical, factors may be responsible for the appearance of metastasis in certain nodes and the lack of metastasis in others, suggesting that cancer is not a local disease, but a systemic disease [39]. Oligometastasis implies a critical interaction between systemically disseminated cancer cells and the host; this concept leads to the notion that confined distant metastasis could be completely cured by local therapy in some cases [40]. Based on these ideas, we believe that lymph node metastasis is both a key step and coincident finding. Thus, an analysis of markers specific for lymph node metastasis may be useful for stratifying prognosis.

Anticancer radiation therapy for head and neck cancer can reduce local recurrence, but not distant metastasis [23,41]. The molecular markers used in the present study are related to the prognosis of other head and neck malignancies, including salivary gland tumors. Although we did not identify the prognostic significance of these molecular markers, immunohistochemical staining revealed high expression levels of c-kit, EGFR and VEGF in the present study. These findings indicate a need for further research on these markers in adenoid cystic cancer.

## Conclusions

Lymph node metastasis was the only factor that was related to recurrence and survival rate following treatment of salivary adenoid cystic cancer. Despite the high expression levels of c-kit, EGFR, and VEGF, these markers were not significantly correlated with recurrence and the prognosis of adenoid cystic cancer. However, the expression levels of c-kit, EGFR, and VEGF exhibited variable changes, suggesting that interrelationships among these proteins may provide approaches for molecularly targeted therapy for salivary adenoid cystic cancer.

## Competing interests

All authors have no competing interests.

## Authors’ contributions

SKL, MSK, and SYN participated in the design of the study, carried out the extraction and analysis of data, and wrote the manuscript. KJC reviewed pathology of the enrolled slides. YSL and SYN performed the critical review of the literature. KJC and SYK assisted in the collection of the clinical data and in reviewing the manuscript. SYN assisted in reviewing the manuscript. All authors have read and approved the final manuscript.
